# Aging and Others' Pain Processing: Implications for Hospitalization

**DOI:** 10.1155/2014/737291

**Published:** 2014-08-31

**Authors:** Alberto Di Domenico, Beth Fairfield, Nicola Mammarella

**Affiliations:** ^1^Department of Psychological Sciences, University of Chieti, Via dei Vestini 31, 66013 Chieti, Italy; ^2^Psychogerontology Center, University of Chieti, Via dei Vestini 31, 66013 Chieti, Italy

## Abstract

*Objectives*. While self-pain perception has been widely investigated in aging, the perception as well as memory of pain in others has received little attention. *Methods*. The study was designed as a cross-sectional behavioral study in which a group of 41 younger and a group of 41 older adults evaluated a series of valenced and pain-related pictures and were later required to recall them. *Results*. We found that older adults judge the stimuli as being less intense compared to their younger counterparts. However, older adults remembered a larger number of pictures with individuals expressing pain compared to pictures with individuals who have neutral or positive facial expressions. *Conclusions*. Older adults may underestimate emotional intensity in others, but they seem to remember painful information in others as well as younger adults. These data are discussed in terms of theories of pain perception and implications for hospitalization.

## 1. Introduction

Older people frequently need to use hospitals or require long stays in residential structures. Consequently, determining different ways of ameliorating their environmental conditions during their illness as well as clarifying psychological factors that may be involved is fundamental. Processing pain in others may be one of them.

In particular, during the last decade, there has been a large increase in interest about the perception of pain in others, especially from a neuroimaging perspective [1]. Studies in this field have found that perception of pain in others involves somatosensory networks that are also common to self-pain perception. This is one of the main reasons why observing other people who are suffering provides information that helps us to understand the affective states of others and rapidly respond to them in a pro-social manner. In the aging population, the study of the perception of pain in others has received little attention. Yet studying the perception of pain in others in aging is relevant in a practical sense as older adults typically show a reduced ability in understanding complex emotional signals in others [2]. In addition, clarifying the effects of age on the perception and memory of pain in others may add useful information about factors influencing prosocial behaviours and eyewitness testimony. Most importantly, it may help clinicians, nurses, and administration to devise policies that include contextual remedies that can be used to reduce pain-related stress and increase patient compliance.

Emotion and aging literature suggests that attention and memory in older adults are particularly sensitive to positive information that aids emotion regulation mechanisms and well-being [3–5]. Consequently, older adults are typically induced to disregard negative information (e.g., pain-related information) and focus on positive information. In addition, neuroimaging studies detected significant age-related changes in brain structures involved in pain processing which may be called on to explain older adults' reduced ability in processing pain-related information [6]. These findings may generally indicate that older adults may be less efficient in encoding and remembering pain-related information compared to younger adults. However, as far as we know, no studies have directly investigated others' pain processing in aging. On one hand, bottom-up theories of others' pain processing, such as the* threat value of pain hypothesis*, suggest that observing pain in others may be perceived as a threatening signal that should lead to immediate avoidance and a reduction in the importance of others' pain [7]. This may be particularly true for older adults who typically show an attentional preference toward positive stimuli rather than negative ones [8, 9]. Consequently, one may expect older adults to underestimate the intensity of pain during online processing of pain in others. In this case, older adults who share a hospital room with a suffering individual may not be immediately affected by pain in others.

On the other hand, top-down theories of others' pain processing suggest that observing others in pain may involve a self-referential processing component that may lead to an increase in the elaboration of others' pain-related stimuli. This may be especially relevant for older adults who typically experience pain more frequently than their younger counterparts [10]. Basically, online and offline processing of pain information may lead to differential patterns of performance especially in aging. Online processing that is more automatic and faster may lead older adults to ignore negative information, while offline processing that is more systematic and slower may lead older adults to regulate information according to their personal history of pain.

In line with this assumption, one may expect older adults to show better memory for pictures expressing pain instead of favouring positive information as previous studies have repeatedly shown [5]. This may lead to an exacerbation of self-pain conditions that may hinder recovering and compliance.

To test these hypotheses, we asked healthy younger and older adults to imagine being in a hospital room with another patient and rate the intensity of the pain perceived in different pictures where male and female individuals expressed pain or happiness or had a neutral expression. Subsequently, without prior notice, we asked them to recall as many pictures as possible. If older adults tend to neglect or diminish others' pain-related information as they usually do with negative information during online processing of pain-related information, we would expect older adults to rate others' pain-related pictures as less intense compared to their younger counterparts. In addition, if this type of material also involves some sort of self-referential processing, we would expect older adults to remember others' pain-related pictures better compared to positive and neutral information.

Our main objective was to start investigating age-related differences in processing pain in others in a group of healthy older adults. We assume that processing pain in the other may be a crucial psychological factor influencing hospital and residential stays.

## 2. Methods

### 2.1. Study Design

The study was approved by the departmental ethical committee. This is a cross-sectional behavioural study on a group of healthy younger and older adults. All participants consented to the analysis of personal data and outcome measures.

### 2.2. Participants

Forty-one healthy younger adults (mean age = 22.66 years, SD = 2.91 years, range = 18–28) and 41 older adults (mean age = 72.66 years, SD = 5.74 years, range = 65–84) were recruited from the Chieti area as participants. Exclusion criteria included self-reports of uncorrected vision or hearing difficulties, a history of psychiatric or neurological disorder, a history of drug or alcohol abuse, a medical history of cognitive deficits (problems with memory, attention, etc.), any serious head injuries or periods of unconsciousness, or if they were taking a prescribed drug with psychoactive properties. Participants' characteristics are reported in Table 1.

Before participating in the experimental session, participants were tested on their general cognitive abilities and self-pain perception. The study was in accordance with the Helsinki Declaration of 1975, as revised in 1983, and the department's ethics committee approved this project. Written informed consent was obtained from each participant. Participants were not payed for their participation.

### 2.3. Pain Measures

Visual stimuli were developed and validated in a pilot study conducted in our lab. Stimuli consisted of 32 pictures, each depicting a different individual (16 male and 16 female actors) with a painful (8 pictures), happy (8 pictures), or neutral expression (16 pictures). These pictures were selected from a larger set of 80 pictures (20 positive, 20 negative, and 40 neutral) that were rated in terms of visual complexity, valence, and arousal on a 9-point scale (from 1 not at all to 9 absolutely) by an independent group of 20 younger and 20 older adults. The 24 pictures were matched in terms of visual complexity and arousal.

Stimuli were presented on a computer monitor with E-Prime 1.2 software (Psychology Software Tools Inc, Sharpsburg, PA).

After completing the informed consent form and the demographic and cognitive screening tests, participants were asked to silently read a brief paragraph describing a hospital room and imagine being there with another patient that could be happy, in pain, or neutral. Subsequently, they rated the levels of pain expressed by individuals in a series of pictures using a visual analog scale (VAS) with the label “no pain” (scored as 0 on 100) at one end and the label “the most intense pain imaginable” (scored as 100 on 100) at the other end.

Each trial began with the presentation of a picture and the scale underneath. Each stimulus remained on the computer screen for 5 seconds during which participants used the mouse to rate the intensity of the pain expressed in the picture by moving a cursor on the VAS. The cursor appeared at a random location on the VAS on each trial. The following trial was separated from the rating by a 1-second white screen. Participants took two practice trials before beginning the experimental session in order to familiarize with the task. Pictures were pseudorandomized in order to avoid seeing more than two consecutive pictures of the same valence. After viewing 32 pictures, participants took a surprise free recall test: participants were asked to free recall all the pictures they could remember. They were informed that this was done to study how people remember their room companions once they leave the hospital.

Specifically, we followed Mather and Knight's procedure [11] and asked participants to write down every picture they could remember (without following presentation order), by describing as many of the details necessary for a hypothetic outsider to univocally identify it within the entire subset of pictures used. Two raters (not psychologists) independently judged recall responses (by assigning one point for each correctly recalled picture), with a third rater being referred to in the event of a disagreement. Only pictures whose written description was sufficiently detailed to allow their univocal identification were classified as remembered.

### 2.4. Statistical Analyses

All statistical analyses were performed with SPSS 13.0 for Windows (SPSS Inc, Chicago, IL). The alpha threshold was set at *P* < 0.05, and post hoc pairwise comparisons were corrected with the Bonferroni procedure.

## 3. Results

### 3.1. Ratings of Pictures

A mixed two-way analysis of variance (ANOVA) with age (2 levels: younger, older adults) as a between-subjects factor and valence (3 levels: pain, positive, and neutral) as a within-subjects factor on pain intensity ratings detected a significant main effect of age (*F*(1,80) = 4.99, *P* < 0.05, *η* ^2^ = 0.06) as older adults rated all pictures as less intense compared to younger adults. There was a significant effect of valence (*F*(2, 160) = 4.79, *P* < 0.001, *η* ^2^ = 0.86) as pain-related pictures were rated as the most intense compared to the other two types of pictures.

Finally, the two-way interaction was not significant (*F*(2,160) = 1.95, *P* = 0.14, *η* ^2^ = 0.02) as both groups showed the same intensity rating trend: pain-related pictures were judged as more intense compared to positive and neutral pictures.

### 3.2. Memory for Pictures

A mixed two-way analysis of variance (ANOVA) with age (2 levels: younger, older adults) as a between-subjects factor and valence (3 levels: pain, positive, and neutral) as a within-subjects factor on mean percentage of recalled pictures detected a significant main effect of age (*F*(1,80) = 10.81, *P* < 0.001, *η* ^2^ = 0.12) as older adults recalled a lower number of pictures compared to younger adults.

The main effect of valence was not significant (*F*(2,160) = 1.85, *P* = 0.16, *η* ^2^ = 0.02). However, the two-way interaction was significant (*F*(2,160) = 6.65, *P* < 0.01, *η* ^2^ = 0.08): LSD post hoc comparisons showed that older adults recalled a lower number of positive (*M* = 11.7, SD = 9.5) and neutral (*M* = 12.1, SD = 6.8) pictures compared to younger adults (positive* M* = 20, SD = 11.6; neutral* M* = 19.8, SD = 8.2). Most importantly, younger and older adults did not differ in terms of number of pain-related pictures (younger adults* M* = 17.6, SD = 14.1; older* M* = 19.5, SD = 10.9) (*P* < 0.05 for all critical comparisons).

Importantly, these effects remained significant even when we used self-pain perception and education as covariates (*P* < 0.05).

Furthermore, to create a better index of the influence of others' pain perception on memory, we computed a pain score. To calculate this index, we considered the proportion of remembered pain-related pictures over the other types of pictures as follows: number of total remembered pain-related pictures divided by [total number of pain pictures + (number of neutral pictures/2) + total number of positive pictures]. We did this to verify whether older adults remembered a greater proportion of pain-related pictures over the total of recalled pictures.

We found that older adults remembered a greater number of pain-related pictures (*F*(1,80) = 17.44, *P* < 0.001, *η* ^2^ = 0.18) out of all remembered pictures.

## 4. Discussion

Pain-related information processing is generally affected by aging. However whether there are age-related differences in all aspects of pain-related information processing still needs to be clarified. Our study is a first attempt to investigate how aging may modulate perception and memory of pain in others. We found that older adults rated the pictures as less intense compared to younger adults. This finding is in line with previous studies which showed smaller intensity effects for affective pictures in older adults compared to younger adults [12]. As explained by emotional intensity theories [13], it is possible that not all stimuli were perceived by older adults as central to their personal goals and thus were generally judged as less emotionally intense. Studies that embraced the socioemotional selectivity approach [4, 14–16] also showed that older adults' emotional response may vary depending on current goals. Generally speaking, these data indicate that older adults' perception of others in pain is not an immediate crucial variable during hospital and residential stay.

Differently, we found memory in older adults was enhanced and reached the level of younger adults' performance when others' pain-related pictures were presented. In this case, age-related differences were nullified. This may be because older adults typically experience pain more frequently than their younger counterparts and are, consequently, more sensitive to and benefit more from contextual pain-related cues that accompany memory formation. Observing others in pain may thus involve a self-referential processing component that enhances the elaboration of others' pain-related stimuli. In addition, according to the arousal-biased competition theory, emotional stimuli, such as people suffering, may have high priority and therefore often win the competition for attentional resources [17]. This arousal-induced attentional focus on items expressing pain results in deeper encoding and subsequently better memory for all participants.

Altogether, results suggest that a combination of perceptual and emotional factors is at work during online processing and remembering of others' pain-related information in aging which may have relevant implications for hospital and residential stays.

However, several limitations must be considered. The first concerns the assessment of pain-related processing. In fact, the assessment of others' pain-related processing conducted in the present study needs to be repeated with a larger number of participants and should take into consideration different dimensions of others' pain. Our set of pictures, in fact, covered different pathologies as backache, heart attack, and headache but, given the low number of the pictures per condition, we were not able to compare across different pain dimensions. It may be also promising to further investigate the role of individual differences, such as comparing suffering versus healthy older adults who are not experiencing pain, investigating gender, and the role of familiarity (in terms of higher frequency of impact) in the type of pain-related information processing. Second, although we sought to explore how individuals process pain information regarding others, it is not clear whether this laboratory-based study elicits the same performance in everyday hospital context. It would be interesting to replicate these data in a real hospital setting and evaluate perception of hospital setting before admission and after hospital dismissal.

## 5. Conclusion

Altogether, this original study demonstrated the specific effects of aging on others' pain processing within a single protocol. While age-related differences seemed to emerge during online processing of affective information, group differences were nullified in memory of others' pain information. This ultimately suggests that others' pain processing is a complex mental ability which must be viewed as a relevant factor affecting hospital stay.

## Figures and Tables

**Table 1:** Sample characteristics.

Characteristic	Younger adults	Older adults
	(*N* = 41)	(*N* = 41)
Age (mean, SD)	22.66 (2.91)	72.66 (5.74)
Gender	20 M 21 F	20 M 21 F
Education (mean, SD)	12.15 (1.9)	6.46 (1.53)
MMSE (mean, SD)		28.1 (1.4)
Forward digit	6.51 (1.23)	4.07 (1.59)
Backward digit	6.12 (1.21)	3.32 (1.06)
Self-pain rating	2.78 (2.58)	4.51 (1.79)
